# Applications of Contrast-Enhanced Ultrasound in Splenic Studies of Dogs and Cats

**DOI:** 10.3390/ani12162104

**Published:** 2022-08-17

**Authors:** Rute Canejo-Teixeira, Ana Lima, Ana Santana

**Affiliations:** 1 Faculty of Veterinary Medicine, Lusófona University, 1749-024 Lisbon, Portugal; 2 Veterinary Teaching Hospital (CA), Faculty of Veterinary Medicine, Lusófona University, 1749-024 Lisbon, Portugal; 3 CECAV-Animal and Veterinary Research Center, Universidade de Trás os Montes e Alto Douro, 5000-801 Vila Real, Portugal; 4 AL4AnimalS-Associate Laboratory for Animal and Veterinary Sciences, Universidade de Trás os Montes e Alto Douro, 5000-801 Vila Real, Portugal

**Keywords:** contrast-enhanced ultrasound, dogs and cats, spleen

## Abstract

**Simple Summary:**

Contrast-enhanced ultrasound (CEUS) is a noninvasive imaging technique that has become a reliable tool for identifying and monitoring lesions in both human and animals. In the last decade, its use in veterinary diagnostic imaging has gained increasing importance, and it can be reliable in everyday clinical practice. However, there is a lack of reviews describing existing CEUS results in the study of splenic lesions, which is of particular importance in dogs and cats. This information is important for validating its efficacy, to facilitate decision making related to sampling procedures and diagnosis, or even as a means to select CEUS as an alternative diagnostic tool in specific cases. Our goal was to review the existing studies of CEUS applications for splenic ultrasound studies in cats and dogs, present these results in a systematic manner, and combine this information into practical guidelines that can be used to help diagnosis and interpretation in both clinical cases and research.

**Abstract:**

Contrast-enhanced ultrasound (CEUS) is an emerging technology in veterinary medicine involving the administration of intravenous contrast agents, and it is increasingly recognized for its high potential as a diagnostic imaging tool for small animals. This exam is easy and quick to perform, safe and reliable, and allows for the differentiation of lesions. It permits the identification of lesions that may require more invasive procedures, from those that can be safely dismissed to those that can be followed-up with ultrasound imaging. Although it has been extensively reviewed for use in human medicine, there is an overall lack of information about the application of this technique for cats and dogs, particularly in splenic studies, which can be particularly important for small animals. The present review describes and summarizes the CEUS applications used for splenic analysis in cats and dogs, providing a basic overview of CEUS technology with examples of common and uncommon features of focal splenic lesions. It also systematically gathers the results obtained for benign and malignant splenic lesions described in the literature, whilst providing guidelines for their interpretation. Furthermore, it presents the advantages of using CEUS for splenic analysis in cats and dogs and the main factors that may influence the quality of the imaging and the accuracy of the diagnosis. This type of knowledge can be used to provide a framework to help veterinarians make informed decisions regarding the use of this emerging technique for splenic lesions, guiding their interpretation of CEUS findings in the splenic ultrasounds of cats and dogs.

## 1. Introduction

### 1.1. CEUS Technique

Contrast-enhanced ultrasound (CEUS) is a noninvasive imaging technique that utilizes contrast agents consisting of microbubbles/nanobubbles of gas to enhance ultrasound imaging, allowing for assessment of the size, shape, texture, and vascularity of several organs [1,2,3,4]. In the last decades, CEUS has become a reliable and efficient tool for the detection, characterization, and monitoring of lesions and pathologies in both humans and animals [4,5]. The fact that it is non-invasive, convenient when compared with other imaging modalities, and has the ability to simultaneously provide real-time anatomical and functional imaging makes this technology a go-to reference in everyday clinical practice, and it is developing an increasingly growing body of research, particularly in human medicine [6,7,8,9]. Paralleling human applications, the use of CEUS in veterinary medicine has been pursued, but, despite some research papers regarding the use of CEUS in small animals and equines [10,11,12,13], veterinary literature is much scarcer and based on a smaller number of cases and diseases. So, although CEUS has been extensively reviewed in human medicine [3,5,6], there is a noticeable lack of reviews concerning CEUS applications in animals, particularly for less studied organs such as the spleen.

### 1.2. CEUS for Splenic Studies

CEUS has been shown to be a particularly valuable tool for splenic analysis, with the ability to determine and monitor alterations in the size, presence, and character of focal lesions, as well as detect specific alterations in its normal vascular pattern [14,15,16,17]. Indeed, in the last 20 years, the use of CEUS techniques for the examination of the spleen in humans has been increasingly pursued and several guidelines [16] and specific studies have arisen [16,17]. However, there are limited studies and reference guides to describe the use of this method for characterizing splenic lesions in veterinary practice. This limitation has likely impaired the diagnosis and characterization of different type of lesions for veterinarians using this technique.

Having a diagnostic algorithm based on current knowledge can be important for the interpretation of results, deciding sampling procedures and diagnosis, or even as a means to select CEUS as an alternative diagnostic tool in clinical and research settings. As a result, the present review aimed at describing the uses of CEUS in the splenic studies of dogs and cats, summarizing the findings obtained in published studies using CEUS to characterize common and uncommon features of focal splenic lesions and organizing them into benign and malignant lesions.

It was also our goal to provide guidance for the interpretation of the CEUS findings using a practical approach, both for data interpretation as well as for decision making regarding follow-up procedures. This type of practical information is pivotal for those deciding to use this technique as a diagnostic tool, as well as for those who wish to have a diagnostic algorithm to guide diagnostic imaging interpretation.

## 2. CEUS Applied for Dogs and Cats

The application of CEUS technology in veterinary research has gained particular momentum in the last decade with the development and improvement of the efficacy of ultrasound contrast agents and specific imaging technologies [18]. One of the main catalysts for the development of in vivo imaging of small animals was the fact that it became a critical tool for drug development and the identification of new clinical targets, as well as the evaluation of drug effects and safety tests in animal models [19,20]. Due to its noninvasive nature, CEUS both reduces the number of experimental animals required for these studies and decreases variability, whilst also permitting a more ethical approach [21,22,23,24,25,26]. Although initially, contrast agents were not used routinely in veterinary patients because of the costs, the growing interest in non-invasive diagnostic procedures and obtaining real-time information has catapulted the use of CEUS in everyday clinical practice [25,26].

### 2.1. The CEUS Technique

CEUS relies on the intravascular injection of specific ultrasound contrast agents which consist of microbubbles containing low-soluble gases that are stabilized by a biocompatible shell [27,28,29,30,31]. The contrast agent microbubbles currently used in everyday clinical applications are known as second-generation agents and are composed of an inert elastic gas surrounded by a stabilizing shell [29,30]. In past years, novel approaches have been developed to improve the contrast enhancing agents used for CEUS, for instance, covering the microbubble shell with targeting ligands that can bind to specific receptors [30] or the development of nanobubbles that are able to cross the vascular endothelium [28,29,30,31,32], hence opening new possibilities for future applications of CEUS studies (Table S1).

**Figure 1:**
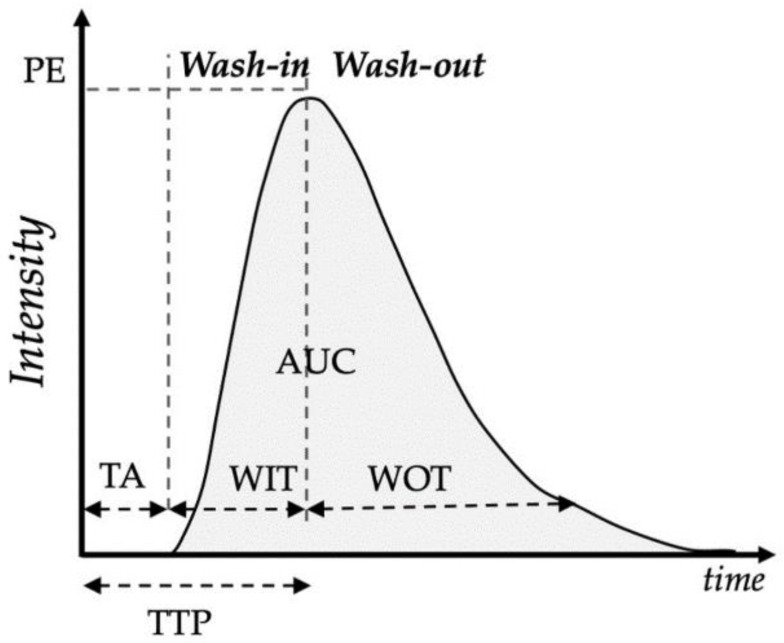
Schematic representation of the CEUS method. A contrast agent is administered as an intravenous injection. The dilution of the contrast agent in the organ of interest is detected downstream by ultrasound imaging. AUC = area under the curve; TA = appearance time; TTP = time to peak; WIT = wash-in time; WOT = wash-out time. Note: parameters may vary depending on the software in use.

The technique is rather simple and requires little more than ultrasound equipment equipped with contrast imaging detection tools, the contrast agent, and saline solution [1,2,33], which makes it more appealing for use in veterinary clinical settings. The dilution and composition of the contrast agent varies according to the manufacturers, the type of organ, and the animal species, and were described earlier for cats and dogs [34]. After injection of the contrast agents, an analysis of the ultrasound intensity curve is performed throughout time, which describes the evolution of the signal enhancement caused by the microbubbles being transported through the organ or tissue of interest, using non-linear imaging modes (contrast modes). The evaluation of the wash-in and wash-out phases of the ultrasound contrast agent (UCA) is completed in mere minutes (Figure 1) [1,2,33].

The enhancement seen on an ultrasound is very noticeable, and the patterns observed are broadly encompassing, including the presence/absence of the contrast, wash-in and wash-out, temporal behavior, perfusion features, vascular anatomy, comparison with the surrounding tissues, and flow direction in lesions, all of which can significantly aid in an accurate characterization of a wide array of pathologies in both human and veterinary medicine [1,2,33]. Both qualitative observations and quantitative measurements can be used as well. Overall, the main diagnostic features are the vascular architecture (evaluated in the early wash-in phase) and contrast enhancement of the lesion compared to the adjacent tissue (time course of the wash-in and wash-out) [1,2,33].

### 2.2. Advantages of CEUS for Cats and Dogs

**Figure 2:**
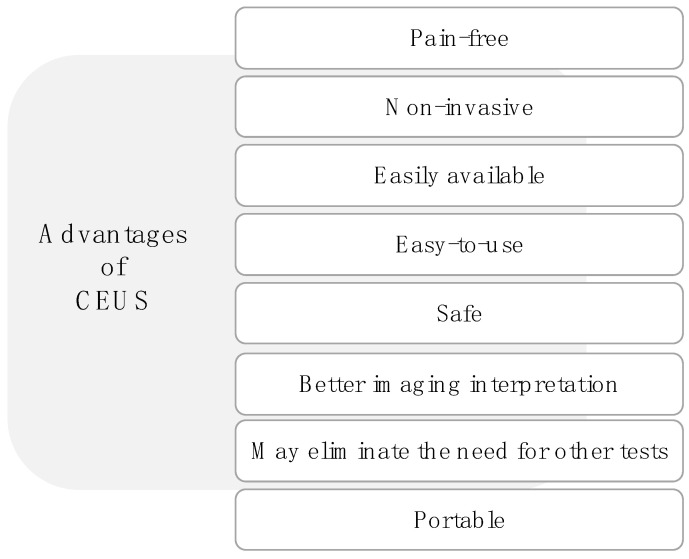
Advantages of CEUS as a diagnostic tool.

As a tool for imaging diagnosis in veterinary applications, CEUS holds several advantages, and it has been described as ideally suited for small animal imaging by several authors [6,24,35,36,37,38,39,40,41] thanks to its very broad range of applications and its high spatial and temporal resolution and low cost [1,2,11,41]. Figure 2 summarizes the benefits of the CEUS technique for cats and dogs.

Overall, CEUS is not only safe and non-invasive, but it also provides reliable quantitative and qualitative measures [1,2,5,18,19,23]. Furthermore, it has been shown to detect alterations that are less easily detected by traditional methods [5,23]. Its portability and easy manipulation also make it easily available for both pet owners and veterinarians [37,38,39,40,41]. When compared to other diagnostic imaging techniques, CEUS also holds several advantages: it is painless, non-toxic, and radiation-free [1,2,14,29], and it has been described as widely available, easy-to-use, and less expensive than other imaging methods [29]. It also provides a clear picture of organs that are more difficult to observe on a survey radiograph and B-mode ultrasound, and abdominal CEUS can eliminate the need for other tests such a CT or an MRI [18,19,24,37,38,39,40,41]. This can be especially helpful in situations where full anesthesia of the patient can represent an unacceptably high risk [38]. It is also noteworthy that the contrast agents used in an abdominal CEUS carry a very small risk of an allergic reaction. In fact, although there is a small risk of allergic reaction for humans, there is no evidence of such reaction in cats and dogs [39,41].

## 3. CEUS in Splenic Studies of Dogs and Cats

Veterinary clinical applications of CEUS focus mostly on the liver [42,43,44,45,46,47], lymph nodes [46], pancreas [47,48,49], kidneys [10,49,50,51], several types of neoplasias [52,53,54,55], portosystemic shunts [56], and last, but not the least, the spleen [15,57,58]. Although the liver, lymph nodes, and superficial neoplasias have been the most commonly studied tissues [26], the diagnosis of splenic alterations is of particular interest due to its involvement in lymphatic, immune, circulatory, and hematopoietic functions [17,38,39,59]. Being one of the most susceptible organs to primary and secondary neoplastic lesions and a wide range of diseases, a detailed assessment of the spleen is of great importance in veterinary medicine, especially in dogs and cats due to their sentimental value [26,39].

### Common and Uncommon Features of Focal Splenic Lesions

The spleen is a parenchymal organ with a superficial location, making it well suited for CEUS examination. When observed by CEUS, the spleen perfusion dynamics differ from the liver, where there is dual blood supply, and they are similar to those seen on a contrast-enhanced CT scan, although with better vascular definition. Usually within the first 20 s after the contrast agent injection, microbubble contrast is seen within the splenic artery and its branches. Subsequently, the opacification becomes inhomogeneous, producing the so-called “zebra” pattern [60,61]. The splenic enhancement becomes homogeneous shortly thereafter, and the overall enhancement lasts only a few minutes [60,61].

Morphologic abnormalities, including focal masses and diffuse alterations (with or without overall splenic enlargement), are common in dogs and cats, as is nodular splenic disease [62,63,64,65,66,67,68]. This has made CEUS a powerful imaging tool to evaluate and detect splenic abnormalities [17]. Because CEUS has been incorporated into everyday clinical practice, malignant diseases such as focal lymphomatous infiltration, metastatic deposits, benign cysts, traumatic fractures, and hemangiomas can be detected and characterized without the need for further imaging [17,58,59,60,61,62,63,64,65,66,67,68]. Hence, in the new era of CEUS, more patients benefit from the radiation-free investigation of splenic pathologies with a high diagnostic accuracy.

## 4. CEUS-Detected Spleen Abnormalities in Dogs and Cats

### 4.1. Benign Diffuse Diseases

As previously stated, several studies in both human and veterinary medicine demonstrate the efficacy of using CEUS in differentiating between malignant and benign focal lesions of the spleen [41,58,69]. On the other hand, when it comes to the use of CEUS in the diagnosis of splenic diffuse alterations, there are few published works that emphasize its advantages over the conventional ultrasound. Examples are a splenomegaly (congestion, splenic hyperplasia/extra medullary hematopoiesis, and inflammatory splenomegaly), an accessory spleen, and an inhomogeneous spleen of unknown causes [70,71,72]. Table 1 summarizes CEUS findings in benign splenic lesions.

Using CEUS, similar to the normal spleen, the enlarged spleen usually shows a homogeneous and marked enhancement of its texture, but this enhancement does not provide more diagnostic differential clues than the conventional ultrasonography [71].

Hyperplastic lymphoid tissue in benign hyperplastic diseases as reactive hyperplasia and splenic hematomas has been reported to be highly vascularized, and in a study with 60 dogs, these benign lesions presented a marked enhancement in CEUS. Nevertheless, this exam was of limited value and histology for confirmation of the benign nature is needed [78,79,80,81].

Leishmaniosis in dogs is known to be responsible for pathological changes in the spleen, namely splenomegaly and diffuse alterations of the eco-structure [82]. In leishmaniotic dogs that show small hypoechoic nodules through the spleen, marbled or moth-eaten lesions (also seen in extramedullary hematopoiesis and lymphoid hyperplasia hyperplasia) in gray scale ultrasound also showed an abnormal diffuse and persistent heterogeneous enhancement at 13, 33, and 60 s using CEUS [79].

CEUS was shown to be useful where there is doubt about the origin of a peri splenic mass (accessory spleen/splenunculis) or of tissue that has arisen post-splenectomy or post-trauma (splenosis) [58]. An accessory spleen has an incidence of around 16% in humans, and it is communally located at the splenic hilum and in the tail of the pancreas and presentes as nodules of variable size (from 1–4 cm) [72,83]. Normally, accessory spleens are easily identified with a conventional ultrasound, but large or atypically located spenunculi can cause diagnostic uncertainty, being misinterpreted as a pathological peritoneal nodule or enlarged lymph node [58,72]. CEUS can confirm that a mass represents ectopic splenic tissue, demonstrating an enhancement pattern typical of normal spleen, which is the persistent late phase enhancement, and differentiating the mass from other lesions such as pancreatic tail tumors, splenic hilar lymph nodes, adrenal lesions, ovarian masses, and metastatic deposits [58,71]. In veterinary medicine, accessory spleens are reported to be rare, but a study in 2010 [83] described the use of CEUS in four dogs where the masses were round to triangular, homogeneous, and hypoechoic, and located between the spleen, the stomach, and the pancreas, with results similar to the ones described above in humans.

In humans, CEUS is also helpful in demarcating and characterizing focal lesions in patients with an inhomogeneous splenic texture [71], as well as to demonstrate splenic involvement in some patients with the underlying diagnosis of malignant lymphoma and increase the diagnostic sensitivity in patients with granulomatous splenic involvement [80]. Nevertheless, in veterinary medicine, to our knowledge, there are no recent studies that demonstrate the clear advantages of using CEUS in the diagnosis or clarification of diffuse inhomogeneous changes in the spleen.

### 4.2. Malignant Splenic Lesions

Although malignant splenic lesions are relatively rare in human medicine [17,84,85,86], in recent years, the importance of correctly diagnosing malignant lesions [84,87,88,89,90], together with the risk of hemorrhage, immune system impairment, and sepsis associated with invasive procedures [84,89] has propelled research into the use of contrast-enhanced ultrasonography to differentiate between malignant and benign lesions in human medicine [69,88,91]. In veterinary medicine, neoplastic splenic lesions are much more common [92,93] and not easily differentiated from benign processes through normal imaging techniques [75,76,77]. Although long-held anecdotal beliefs, such as the presence of a cavitated splenic mass being indicative of neoplastic lesions, have proven to be unfounded, a diagnostic ultrasound continues to be a critical part of managing splenic lesions, as well as planning surgical approaches [94]. The fact that survival time after surgical intervention for those with benign lesions is significantly longer than those with neoplastic lesions [95,96] has profound implications on the decision-making process in the management of a case and whether euthanasia might be considered [97,98,99,100,101,102]. In an attempt to classify splenic lesions, ultrasound guided cytology is often the first approach [57,99,100,101], but this does not always provide a clear answer as to the nature of the lesion [77], and histopathological evaluation, considered the gold standard for the diagnosis of splenic lesions [57,96], must still be performed. This has resulted in the search for alternative forms of classifying splenic neoplastic lesions through the use of CEUS.

In human medicine, studies have shown that the use of CEUS does allow for the identification of malignant vs. benign splenic lesions, the former showing hypo-enhancement in the parenchymal phase independently of the enhancement in the arterial phase, with a faster wash-out rate compared to the surrounding normal splenic tissue [17,68,103,104,105,106]. In veterinary medicine, besides there being a greater range of histopathological changes in the spleen, they are more frequently found than in human medicine [91,92,93]. This has resulted in the study of a wider variety of neoplastic lesions and their CEUS characteristics. Table 2 summarizes CEUS findings in malignant splenic lesions.

Rossi et al. [77] studied 18 dogs and cats with 8 different types of malignant lesions and found that although the wash-in and peak phases showed some variations, all of them had hypo-enhanced wash-out phases. These results were confirmed in two other studies with 29 [78] and 16 [75] dogs, which included malignant lesions not observed in the original study: osteosarcoma, leiomyosarcoma [75], and plasmacytoma [78].

When looking at the characteristics of individual malignant lesions, malignant lymphomas were found to have the highest peak-intensity and AUC while also presenting the fastest wash-in and wash-out phases [56], while lympho-sarcomas showed only early wash-in and wash-out phases [77]. Hemangiosarcoma is the splenic neoplasia that has proven to be the hardest to diagnose definitively, presenting challenges even for histopathology. CEUS has proven unsuccessful in differentiating malignant hemangiosarcoma from a benign hematoma [76], and it has been suggested that the well-vascularized tissue present in areas of hemorrhage may also exist in areas of high cell proliferation, as in a hemangiosarcoma [104]. This maybe the reason why some hemangiosarcomas, in contrast with other malignant lesions, show hyperenhancement [76,78]. This could also present an explanation as to why aberrant vessels with corresponding high peak intensities have been found in hemangiosarcomas [74]. One study looking at five cases of malignant splenic lesions, of which there were hemangiosarcomas, found that although hypo-enhancement in the wash-out phase could not differentiate malignant from benign lesions, the presence of tortuous vessels feeding the lesion could [74]. This reinforces the possibility that differentiation between hematoma and hemangiosarcoma should focus on vascular CEUS patterns, a reflection of how the lesions are perfused [105], as opposed to contrast enhancement in any phase.

### 4.3. Guidelines for Interpreting the CEUS Findings of Splenic Masses

The introduction of CEUS has come to play an important role in the field of diagnostic imaging of splenic lesions, some of which are frequently identified during routine examination, especially in older animals. Though splenic pathology is often clinically silent, when compared to other abdominal organs, it can also be encountered by ultrasound routine exams, which usually provide valuable additional information about splenic abnormalities. Nonetheless, the characterization of focal splenic lesions by ultrasound can be quite difficult. Many lesions are often incidental findings and represent a diagnostic challenge. The conclusive diagnosis of various splenic pathologies is also difficult for the untrained eye and often can only be obtained by follow-up histopathological analysis. Overall, there is a lack of guidelines for CEUS splenic findings that may be used by veterinarians to make informed decisions and to guide them on their interpretation of CEUS findings in the splenic ultrasounds of cats and dogs.

**Figure 3:**
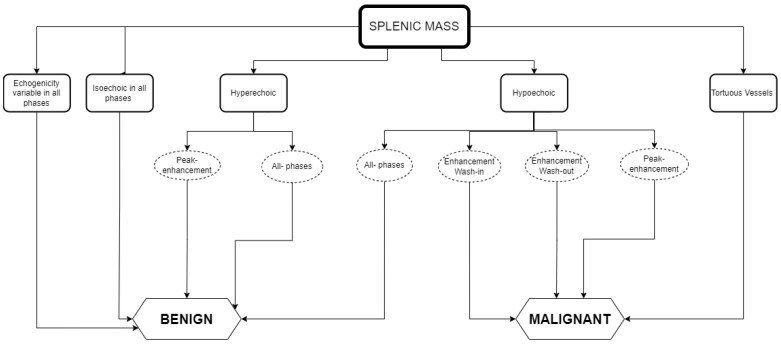
Guidelines for the CEUS data interpretation of splenic masses in cats and dogs: the relationship between the data obtained in CEUS and the type of splenic mass detected.

Here, we combined the results obtained for malignant and benign splenic masses in CEUS findings for dogs and cats as a guide to help elaborate a list of differential diagnoses. In this way, lesions that may require diagnostics can be differentiated from those that can be safely dismissed or followed-up with regular ultrasound imaging. Figure 3 depicts the several interpretations that can be obtained when identifying splenic masses using CEUS, which are gathered in practical guidelines.

**Figure 4:**
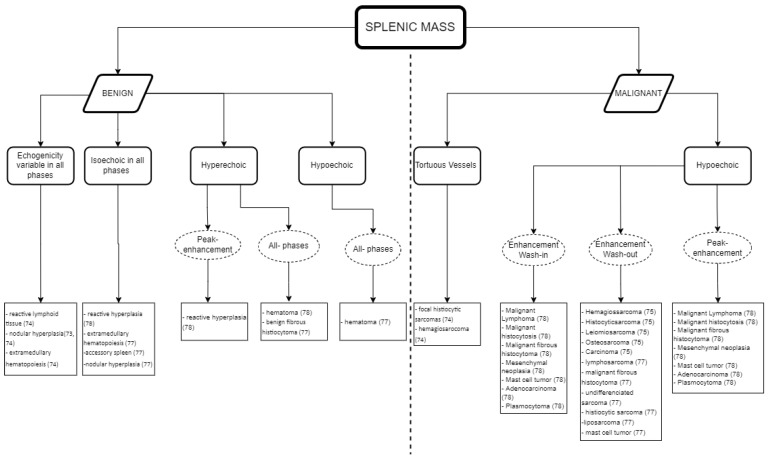
Guidelines for the CEUS data interpretation of splenic masses in cats and dogs: the expected diagnosis for benign and malignant splenic masses according to the data obtained in the CEUS examination.

Whilst Figure 3 shows an interpretation guide for CEUS findings in suspicious splenic masses found in a CEUS exam. Figure 4 describes the type of diagnosis expected for benign and malignant splenic masses, according to the CEUS analysis results, using the data obtained in the literature described above in Table 1 and Table 2, and aimed at facilitating the decision-making process about whether to follow other diagnostic procedures such as cytology and histopathology.

As a practical approach, Figure 3 and Figure 4 can both be used for data interpretation, as well as for decision making regarding follow-up procedures. Although benign lesions are slightly more common than malignant lesions, an accurate diagnosis is often difficult and/or has a wider differential diagnosis. The diversity of the CEUS data obtained in spleen exams can frequently provide valuable additional information to narrow the differential diagnosis and, particularly, to pinpoint the lesions that are likely to be benign from those that may be malignant.

The summary figures provided can be used in clinical practice when faced with a splenic lesion on a B-mode ultrasound and when a CEUS scan is subsequently performed to clarify the nature of the lesion. Figure 3 can be followed after CEUS has classified the vascular pattern to establish the probability of the lesion being malignant or benign. Figure 4 can then be consulted to establish a list of possible differentials for the identified lesions. In this way, the decision-making process for recommending advanced and invasive diagnostics or adopting a wait and see approach can be rationalized.

## 5. Important Factors to Consider When Using CEUS for Splenic Studies

**Figure 5:**
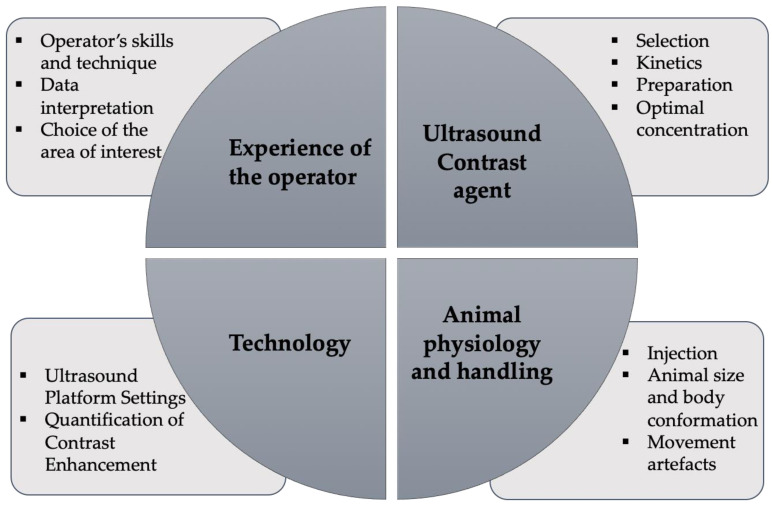
Factors that can influence the quality of a CEUS analysis in veterinary applications.

Despite its several advantages, CEUS can present some limitations which can be of importance when evaluating the spleen. For instance, air can disrupt ultrasound waves [1,2,3,4,5,25,59], and so a gas-filled and distended gastrointestinal tract may pose some difficulties in CEUS evaluations. Larger or obese small animals also present a particular challenge, since larger tissue masses may abate the sound waves [11,25,59]. Another often referred limitation is the fact that CEUS is not suitable for looking at several organs at once, with just one region of the body being examined per injection [1,2,3,4,5,11,25]. Furthermore, there is often a limited view of the relevant organ or lesion, which may pose some challenges. Smaller lesions are also more difficult to detect [11,25,59]. It is also noteworthy that the efficacy of the exam depends on several global factors which may alter the quality and accuracy of the CEUS analysis, particularly when using it on small animals [1,5,23,24,27,28,29,30,31,32,33]. These main factors are summarized in Figure 5.

We have overall grouped these factors into four categories: the animal physiology and handling, the contrast agents used, the technology, and experience of the operator involved. Several publications refer the need for standardized procedures concerning these main factors in order to avoid result hampering, whilst improving the reproducibility and accuracy of the CEUS analysis [1,2,3,4,5,11,25]. In short, a rigorous step-by-step protocol is normally advised, ensuring control of (1) the preparation and the use of the ultrasound contrast agent (concentration and type of microbubbles used), (2) the animal physiology and handling, (3) the injection (dose and procedure), (4) the ultrasound platform settings, (5) data acquisition and evaluation, and (6) the operator’s skills and experience. In addition, ethical recommendations must also be considered.

## 6. Conclusions

CEUS has been clearly shown to be a valuable technique to detect and characterize splenic lesions in dogs and cats. Besides being safe and easy to perform, it presents a multitude of advantages for small animals that appeal to both pet owners and veterinarians alike. CEUS is also indicated in confirming the nature of suspected splenic abnormalities, bringing advantages when compared to other techniques.

Although there is still a scarcity of CEUS splenic studies when compared to other organs, the number of reports has been rapidly rising and confirms its potential. This growing body of evidence suggests that this technique has a strong potential to be more widely used as a first-line or problem-solving tool in the future for routine clinical approaches.

## Figures and Tables

**Table 1:** Summary of CEUS findings in benign splenic lesions.

Diagnosis of Malignancy	Diagnostic Procedure	n	Age (Mean)	Species	Sex	CEUS Findings	Contrast Medium	Stats.	Ref.
Nodular hyperplasia	Cytology *	20	*	D	*	Isoechoic wash-in, hypoechoic peak enhancement, and anechoic wash-out	Sulphur hexafluoride	CI 95% *	[73]
Nodular hyperplasia,	One or a combination of cytology and histopathology *	7	10.1 yrs	D	M/F *	Variations in all phases *; no tortuous vessels seen	Sulfur hexafluoride	Sensitivity, specificity, and accuracy	[74]
extramedullary hematopoiesis,		4							
one reactive lymphoid tissue		1							
Nodular hyperplasia	Histopathology (6) and cytology (2)	8	8.5 yrs	D	M (5), F (3)	Isoechoic vascular and hypoechoic parenchymal phases	Perflubutane	2-tailed Fisher’s exact test, sensitivity, and specificity with 95% CI	[75]
Hematoma	Histopathology	2	10.5 yrs	D	M (1), F (1)	Heteroechoic vascular and hypoechoic parenchymal			
Extramedullary hematopoiesis	Histopathology	2	9.5 yrs	D	M (2)	Isoechoic vascular and hetero-isoechoic parenchymal			
Granuloma	Histopathology	1	10	D	M (1)	Isoechoic vascular and hypoechoic parenchymal			
Hematoma	Histopathology	5	10.6 yrs	D	M, F *	Not able to differentiate from hematomas	Perfluoropropane	Unpaired 2-tailed *t*-test	[76]
Hematoma with hyperplasia		2							
Nodular hyperplasia	Histopathology and cytology *	6	10 yrs	D, C *	M, F *	Isoechoic in all phases without tortuous vessels	Sulfur hexafluoride	None reported	[77]
Extramedullary hematopoiesis		2							
Hematoma		1				Hypoechoic in all phases			
Benign fibrous histiocytoma		1				Hyperechoic in all phases with dense vessels			
Accessory spleen (splanunculus)		1				Isoechoic			
Reactive hyperplasia	Histopathology and cytology *	8	9.6 yrs	D	M, F *	Isoechoic and hyperechoic in wash-in and peak enhancement and variable in wash-out	Sulphur hexafluoride	Fisher’s exact test and odds ratios with 95% CI	[78]
Nodular hyperplasia		10				Variable in all phases			
Extramedullary hematopoiesis		6				Generally isoechoic in all phases			
Hematoma		3				Hypoechoic and hyperechoic in all phases			
Leishmaniosis (normal spleen)	Cytology	22	4.9 yrs	D	M, F *	Variable in all phases, depending on architecture of the spleen, and no difference in quantitative measurements were found	Sulphur hexafluoride	ANOVA	[79]

* details unspecified by authors; yrs = years, D = dog, C = cat, M = male, F = female, CI = confidence interval.

**Table 2:** Summary of CEUS findings in malignant splenic lesions.

Diagnosis of Malignancy	Diagnostic Procedure	n	Age (m)	Species	Sex	CEUS Findings	Contrast Medium	Stats.	Ref
Focal histiocytic sarcomas	One or combination of cytology and histopathology *	2	10.1 yrs	D	M/F	Hypoenhanced in parenchymal phase; tortuous vessels in all phases	Sulfur hexafluoride	Sensitivity, specificity, and accuracy	[74]
Hemagiosarocoma		3							
Hemagiosarocoma	Histopathology	8	12 yrs	D	M (3), F (5)	Hypoenhanced in late vascular phase	Perflubutane	2-tailed Fisher’s exact test, sensitivity, and specificity with 95% CI	[75]
Lymphoma	Cytology	3	5 yrs	D	M (1), F (2)				
Histiocyticsarcoma	Histopathology	2	9.5 yrs	D	M (1), F (1)				
Leiomyosarcoma	Histopathology	1	14 yrs	D	F (1)				
Osteosarcoma	Histopathology	1	12 yrs	D	F (1)				
Carcinoma	Histopathology	1	11 yrs	D	F (1)				
Lymphosarcoma	Histopathology and cytology *	7	10 yrs	D, C *	M, F *	Hypoechoic in wash-out phase (late vascular phase)	Sulfur hexafluoride	None reported	[77]
Hemangiosarcoma		4							
Malignant fibrous histiocytoma		2							
Undifferentiated sarcoma		1							
Histiocytic sarcoma		1							
liposarcoma		1							
Mast cell tumour		1							
Metastasis *		1							
Hemangiosarcoma	Histopathology	11	10.6 yrs	D	M, F *	Not able to differentiate from hematomas	Perfluoropropane	Unpaired 2-tailed *t*-test	[76]
Hemangiosarcoma	Histopathology and cytology *	10	9.6 yrs	D	M, F *	Hypoenhancement in wash-in and peak enhancement and wash-out phase	Sulphur hexafluoride	Fisher’s exact test and odds ratios with 95% CI	[78]
Malignant lymphoma		6							
Malignant histiocytosis		5							
Malignant fibrous histiocytoma		1							
Mesenchymal neoplasia		3							
Mast cell tumour		2							
Pancreatic adenocarcinoma metastasis		1							
Plasmocytoma		1							

* details unspecified by authors; yrs = years, D = dog, C = cat, M = male, F = female, CI = confidence interval.

## Data Availability

Not applicable.

## Supplementary Materials

The following supporting information can be downloaded at: https://www.mdpi.com/article/10.3390/ani12162104/s1, Table S1: Summary of CEUS contrast agents.
