# Supplementary material for: Applications of Contrast-Enhanced Ultrasound in Splenic Studies of Dogs and Cats
Source: Animals (Basel). 2022 Aug 17;12(16):2104. doi: 10.3390/ani12162104 (PMC9404716; doi:10.3390/ani12162104)
Supplement: Supplementary file 1 [file animals-12-02104-s001.zip › animals-1794798-supplementary.pdf]

Table S1: Summary of CEUS contrast agents.

Type	Description	Examples
First generation	First-generation ultrasound contrast agents contained microbubbles of air that were dissolved in blood when exposed to acoustic pressure in the ultrasound field and were therefore present in the bloodstream for a limited time. The generation of US contrast agents is categorized according to the type of gas within the microbubble shells.	**Levovist** (Bayer Schering Pharma, Berlin, Germany): consisted of air within a shell of galactose microparticles (99.9%)/palmic acid (0.1%).
Second generation	In second-generation contrast agents the air in the microbubbles is replaced by a more inert and slowly diffusing gas such as perfluorocarbon, nitrogen gas or sulfur hexafluoride stabilized in a phospholipid membrane. The bubbles oscillate when exposed to the ultrasound beam (they are being compressed by the effect of positive pressure created by the ultrasound waves and they expand in the negative pressure phase). The compression of the gas is greater than expansion which creates a non-linear response (echo). This increases vascular contrast.	**SonoVue** (Bracco, Milano, Italy): consists of sulfur hexafluoridewithin a phospholipid shell<br><br>**Sonazoid** (GE Healthcare, Oslo, Norway - as of 2020 approved in Japan, Korea, Taiwan, and China): consists of perfluorobutane within a hydrogenated egg phosphatidylserine (HEPS) shell<br><br>**Definity** (marketed in North America as Luminity by Lantheus Medical Imaging, North Billerica, MA, USA): consist od octafluoropropane gas within a lipid shell<br><br>**Optison** (GE Healthcare, Princeton, NJ, USA): consists of octafluoropropane within an albumin shell
